# Assessing the effectiveness of tumour board education among medical students: a pre-post-test analysis

**DOI:** 10.3332/ecancer.2024.1727

**Published:** 2024-07-10

**Authors:** Fatima Shaukat, Tayyab Siddiqui, Yumna Ahmed, Muneeba Khan, Mariam Fahim, Asna Noor, Agha Muhammad Hammad Khan, Ahmed Nadeem Abbasi

**Affiliations:** 1 Radiation Oncology, Cyberknife and Tomotherapy Center, Jinnah Postgraduate Medical Center (JPMC), Karachi 75510, Pakistan; 2 Surgical Oncology, Dow University Hospital (OJHA Camp), Karachi 75300, Pakistan; 3 Jinnah Sindh Medical University (JSMU), Karachi 75510, Pakistan; 4 Radiation Oncology, Mc Gill University, Quebec H3A 0G4, Canada; 5 Radiation Oncology, Aga Khan University, Karachi 74800, Pakistan

**Keywords:** students, medical, tumour boards, education

## Abstract

**Introduction:**

Multidisciplinary tumour boards (MDTs) play a vital role in providing high-quality cancer care. In Pakistan’s compromised healthcare system, there is a lack of tumour board establishment. To bridge this gap, we aimed to enhance medical education by exposing medical students to the processes and advantages of MDTs early in their careers through conducting a mock tumour board. This approach seeks to provide students with a practical understanding of cancer care and the collaborative decision-making involved in managing cancer patients.

**Methodology:**

A session took place at Jinnah Postgraduate Medical Centre in May, 2023, with participants voluntarily agreeing to attend. This session comprised six components: a Pretest questionnaire, a didactic lecture on the concept of tumour boards, an interactive group discussion following the lecture, a simulated tumour case presentation, a workshop simulating a tumour board scenario and a Posttest questionnaire.

**Results:**

A total of 80 participants were included in the study. The mean age of study participants was 22. Among these, 36 (45%) were in their final year, 34 (42.5%) in their fourth year and 10 (12.5%) in their third year. While the majority of students possessed a fundamental understanding of tumour boards, they lacked awareness regarding their importance, implementation and procedural aspects involved. Before the mock tumour board, 53 (66.3%) students were unfamiliar with tumour board procedures; post-workshop, all 80 (100%) gained awareness of the process. Additionally, the study showed a positive shift in perceptions regarding the cost-effectiveness of tumour boards. Initially, 44(55%) students responded with ’maybe’ regarding tumour board’s cost-effectiveness, but after training, 64 (80%) perceived it as cost-effective from patient’s perspective. Moreover, students’ overall pretest score was 66.5%, and posttest was 94.62%., showing an overall difference in knowledge of 28%.

**Conclusion:**

The mock tumour board workshop successfully heightened students’ understanding of tumour board procedures, positively shaped their views on cost-effectiveness, and resulted in a noteworthy enhancement of their knowledge scores. Organising similar workshops for undergraduates offers a practical approach to bridging the current gap in the establishment of tumour boards in the future in Pakistan.

## Introduction

A tumour board is a regular meeting held for medical professionals from different specialties to discuss cancer cases and share their specific knowledge regarding the cases. Site-specific multidisciplinary tumour boards (MDTs) are essential for comprehensive cancer care, as validated globally. Tumour board practice in Pakistan is still developing, especially in public sector hospitals. Healthcare workers are voluntarily working to establish these teams in various institutes, facilitating the formation of tumour boards despite numerous challenges [1]. Traditionally, tumour boards bring together specialists from fields such as medical oncology, radiation oncology, surgery, radiology and pathology to provide a multidisciplinary approach to cancer diagnosis and treatment.

Educating medical students about these boards is crucial for improving future cancer care. For the past few years, these students have been voluntarily aiding in tumour board establishment in public sector hospitals. Currently, the most exposure that medical students receive regarding cancer cases is mostly just a month-long rotation in the oncology department. Unfortunately, this limited duration is inadequate for them to truly comprehend the intricate reality of this disease. There are significant medical education disparities among medical students when it comes to oncology, ranging from an inadequate grasp of fundamental management principles to a limited understanding of long-term treatment side effects, survivorship, radiation oncology and palliative care [3, 4]. Therefore we aim to bridge this gap by sensitising the medical students from a very early stage in their careers. Another approach for this is the mock tumour boards which aim at a teaching method that is gaining popularity for its contribution to improving the knowledge and skills of medical professionals engaged in cancer care.

One of the major advantages of mock tumour boards is that they bridge the gap between theoretical knowledge and practical application. Medical students and professionals can learn how to approach complex cancer cases, evaluate different treatment options and consider multidisciplinary input in a setting that mirrors real clinical scenarios. This not only prepares them for the challenges they will face in clinical practice but also promotes a holistic understanding of cancer care that goes beyond the expertise of any single medical specialty [2].

To address this deficiency, various initiatives have emerged. Student-led oncology interest groups provide opportunities for students to explore their interest in oncology, engage in relevant activities and gain exposure to this field. Participating in tumour board shadowing allows students to observe real tumour board sessions, gaining insight into the collaborative decision-making process and the multidisciplinary approach to cancer treatment [5, 6].

Therefore, we decided to conduct a mock tumour board to increase awareness and sensitisation among undergraduate medical students at an early stage of their education. This approach ensures that as they advance in their careers, they will possess the essential medical knowledge and the correct approach to managing this terminal illness. By conducting mock tumour board activities, we aim to provide practical experience and a comprehensive understanding of the operational aspects of tumour boards, equipping students with the necessary skills and knowledge for their future roles in multidisciplinary cancer care teams.

## Methodology

A single-day session was conducted in May 2023 at Jinnah Postgraduate Medical Centre, a tertiary care hospital in Karachi, Pakistan, and participants were recruited from the Jinnah Sindh Medical University student body. Institutional Ethical Review Committee approval was obtained. All those who agreed voluntarily to attend the session were eligible to participate in this study. The aim of the session was to raise awareness among undergraduate medical students about tumour boards and enhancing their understanding of the subject. The entire single-day session consisted of the following six components:

**Pre-test:** The session commenced with the administration of the pre-test consisting of 20 questions related to basic knowledge and the importance of tumour boards.

**Lecture:** A didactic lecture regarding the concept of tumour boards was delivered by an oncologist consultant. The lecture introduced the participants to the concept of tumour boards, their functions, and their importance in multidisciplinary cancer care.

**Interactive group discussion:** Following the lecture, an interactive group discussion was facilitated by the team members. This discussion allowed participants to engage in dialogue, share their thoughts and ask questions, effectively consolidating the information presented in the lecture.

**Simulated tumour case presentation:** A simulated tumour case presentation was conducted to demonstrate the practical application of tumour boards in real clinical scenarios.

**Workshop:** A workshop was organised, during which 40 out of the 80 participants voluntarily stepped forward to participate. These participants were divided into four teams, each consisting of ten students. Each team was tasked with presenting a tumour case, simulating a tumour board scenario. This hands-on activity encouraged active participation and critical thinking.

**Post test:** The interactive session concluded with the administration of the post-test to gauge changes in participants’ knowledge following the session.

### Data analysis

The data were entered and analysed in SPSS version 23.0. Demographics for categorical variables such as mock items were reported as frequencies and percentages. For numerical variables such as age, pre and post test scores were reported as mean ± standard deviation. The frequency of correct responses was calculated by adding the sum of the mock items. The normality factor on numerical variables was seen by the Shapiro Kolmogorov Test. To check the significant differences between pre and post mock test paired *t* test was applied. A *p*-value less than 0.05 was taken as significant.

## Results

A total of 80 participants were included in the study of whom, all students completed both the pretest and the posttest, which indicated a response rate of 100%. The average age of the study participants was 22. Among these, 36 (45%) were in their final year, 34 (42.5%) in their fourth year and 10 (12.5%) in their third year (Table 1). While most students had a basic understanding of tumour boards, there was a lack of awareness regarding their significance, implementation and procedural aspects.

**Figure 1.**
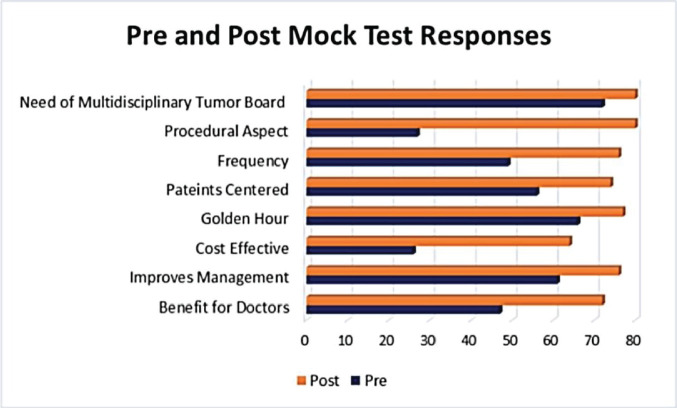
Pre and post correct responses of the mock test.

Before the mock tumour board, 53 (66.3%) students were unfamiliar with tumour board procedures; post-workshop, all 80 (100%) gained awareness of the process. Initially, 72 (90%) of the participants agreed that there was a need to establish tumour boards, after the workshop all 80 (100%) agreed to the establishment of tumour boards. In terms of meeting preferences, initially, 49 (61%) and 25 (31%) of students favoured ’weekly’ and ’monthly’ meetings, respectively. Following the workshop, a significant shift occurred, with 76 (95%) students favouring ’weekly’ meetings (Figure 1).

Tumour boards, assessed during the workshop, were found to be both patient-centered and cost-effective. In the initial assessment, 56 (70%) participants perceived tumour boards as patient-centered, a perception that increased to 74 (92%) post-training. Before the workshop, 66 (82%) participants believed tumour boards were instrumental in saving crucial hours for tumour patients, and after the session, 77 (96%) participants affirmed this viewpoint. Notably, the study revealed a positive shift in perceptions regarding the cost-effectiveness of tumour boards. Initially, 44 (55%) students expressed uncertainty about cost-effectiveness, but after training, 64 (80%) students viewed tumour boards as cost-effective from the patient’s perspective. Primarily, 61 (76%) students agreed that tumour board meetings improved the mortality rate of cancer patients, and post-training, this agreement rose to 76 (95%) students.

Moreover, the tumour board significantly enhances a doctor’s capacity to make decisions regarding the improvement of a patient’s treatment plan. Initially, 47 (59%) students regarded it as ‘most effective’ for doctors. However, after the training session, this perception increased, with 72 (90%). Furthermore, during the training, an overwhelming majority of students 79 (99%) understood that patients have derived benefits from the tumour board, as it effectively saved time, money and energy for the patients overall (Table 2).

The comprehensive pre-test score stood at 66.5%, while the post-test score reached 94.62%. Consequently, the mock tumour board exercise demonstrated a 28% more significant impact on the understanding of undergraduate students overall (Table 3).

## Discussion

In modern healthcare, effective communication and collaboration are essential. This holds particularly true for the constantly evolving field of oncology [7]. Oncologists regularly engage in evaluative discussions with their peers to ensure optimal patient outcomes. Tumour boards play a crucial role in facilitating interdisciplinary expert discussions and providing tailored therapeutic recommendations based on individual patient characteristics [8, 9]. Despite their importance, medical students frequently lack exposure to such interdisciplinary discussions, as tumour boards are not currently integrated into medical curricula. Additionally, this exposure aids students in recognising the significance of considering diverse perspectives and engaging in collaborative decision-making, especially within the context of cancer care [10, 5].

In Pakistan, the quality of healthcare is affected by the challenges of being a low- to middle-income country, which also impacts the establishment of tumour boards in public sector hospitals. Consequently, there is a need to educate undergraduate students about tumour boards, enabling them to contribute to the formation of MDTs by addressing logistical and scientific issues. To achieve this, students should gain a comprehensive understanding of the practical aspects of how MDTs are conducted, as the practical application can enhance theoretical knowledge.

Our study focused on assessing the impact of an in-person workshop designed to enhance medical students’ awareness of the significance of tumour boards. The approach involved active participation through simulations of mock tumour boards and role-play exercises. Past research indicates that role-play, especially centered on communication, serves as a valuable tool to heighten medical students’ interest in clinical education, leading to improved learning outcomes [11, 12]

According to the results obtained from the pre-test and post-test questionnaires, there was a significant improvement in the participants’ knowledge and perception of tumour boards after completing the workshop. In particular, there was a notable shift in their understanding of the benefits of tumour boards from a cancer patient’s perspective. In our study, 80% of students considered tumour boards as cost-effective from the patient’s standpoint, and 95% believed that it enhances mortality outcomes. These findings align with studies conducted in developing nations, affirming the cost-effectiveness and positive influence of tumour boards on patient outcomes [13, 14]. For instance, a prospective cohort study by Brandão *et al* [15] in Mozambique, Africa, demonstrated a 53% reduction in mortality among breast cancer patients after the implementation of an MDT, highlighting its cost-effectiveness as an intervention.

Remarkably, there was a notable change in participants’ understanding of the benefits for doctors associated with tumour boards. Before the session, only 58.8% acknowledged that tumour boards were most beneficial for physicians, but this figure increased to 90% in the post-workshop test. This highlights that a significant portion of students (41.2%) were unsure or unaware of the advantages for doctors before the workshop. Additionally, our pre-test findings revealed that a majority of participants (66.3%) had limited prior knowledge of how tumour board’s operate. This knowledge gap may be attributed to shortcomings in the existing medical education curriculum. Many medical schools in developing countries struggle to provide comprehensive insights into clinical oncology, often lacking opportunities for significant patient interaction, extended case follow-ups, or exposure to multidisciplinary care [16].

Alongside increasing awareness, facilitating and encouraging medical students to attend genuine tumour board meetings helps in improving their understanding of cancer care [17]. A study conducted by Tsui *et al* [6] and few other studies showed that medical students reported better educational learning and knowledge of multidisciplinary care in a mentored tumour board shadowing program as compared to their usual clinical experience [12, 18].

On a larger level, involving medical students in tumour board meetings can affect not only their education and career decisions but positively impact the community at large. Their involvement may lead to the long-term establishment of site-specific tumour boards in teaching hospitals and clinics, particularly where such boards are currently absent, as is often the case in public hospitals in Pakistan [19]. Medical students can also play a pivotal role in organising virtual tumour boards and facilitating communication between specialists from different disciplines to establish regular multidisciplinary meetings. This could lead to increased attendance and better patient care as well as increase the accessibility and feasibility of tumour boards [20].

Our study acknowledges limitations, including a small sample size and being conducted at a single center and only short term effect was studied. However, we mitigated these constraints by employing a diverse teaching methodology within a single day to ensure the delivery of the most comprehensive knowledge possible.

## Conclusion

Providing early exposure to fundamental concepts of cancer management and interdisciplinary care can boost students’ confidence in oncological settings and stimulate interest in pursuing a career in oncology. This proactive approach has the potential to address the shortage of clinical oncologists, especially in developing countries grappling with a significant cancer burden. By fostering early sensitisation, we aim to inspire the next generation of medical professionals to actively contribute to the field of oncology and improve patient care in regions facing unique healthcare challenges.

## Conflicts of interest

The authors report no conflicts of interest in this work.

## Funding

No funding was received for this article.

## Author contributions

Fatima Shaukat and Tayyab Siddiqui: idea, conception or design of the work; or the acquisition, analysis, or interpretation of data for the work, final approval of the version.

Muneeba Khan, Mariam Fahim and Asna Noor: manuscript writing and drafting the work or revising it critically for important intellectual content.

Yumna Ahmed: interpretation of data of work, final approval of the version to be published.

Agha Muhammad Hammad Khan: drafting and revision of work.

Ahmed Nadeem Abbasi: accountable for all aspects of the work in ensuring that questions related to the accuracy or integrity of any part of the work are appropriately investigated and resolved.

## Figures and Tables

**Table 1.** Baseline demographics.

Baseline characteristics	Mean or median (IQR) *n* (%)
Age; Mean ± SD	22 ± 1.06
Age; Median (IQR)	22 (21–23)
Year of MBBS	
Third year	10 (12.5%)
Fourth year	34 (42.5%)
Final year	36 (45%)

SD: Standard deviation; MBBS: Bachelor of Medicine and Bachelor of Surgery

**Table 2.** Descriptive of pre and post-test responses of the mock items.

	Pre-score	Post-score
	*n* (%)	*n* (%)
Have you heard of the concept of tumour board?		
No	8 (10%)	-
Yes	72 (90%)	80 (100%)
Do you know why there is a need to establish tumour boards?		
No	8 (10%)	-
Yes	72 (90%)	80 (100%)
According to you, why were tumour boards not established earlier?		
Lack of awareness	22 (27.5%)	13 (16.3%)
Lack of funding	2 (2.5%)	3 (3.8%)
Lack of interest	3 (3.8%)	1 (1.3%)
Political factors	-	-
All above	53 (66.3%)	63 (78.8%)
Do you think establishment of tumour boards is a necessity?		
No	-	-
May be	5 (6.3%)	1 (1.3%)
Yes	75 (93.8%)	79 (98.8%)
Do you think tumour boards are patient centered?		
No	5 (6.3%)	6 (7.5%)
May be	19 (23.8%)	-
Yes	56 (70%)	74 (92.5%)
What are the benefits of conducting a tumour board?		
Early diagnosis	2 (2.5%)	-
Prompt referrals	1 (1.3%)	-
Time management	-	-
Treatment options	4 (5%)	1 (1.3%)
All	73 (91.3%)	79 (98.8%)
Do you know how tumour boards are conducted?		
No	53 (66.3%)	-
Yes	27 (33.8%)	80 (100%)
Tumour patients have a race against time; do you think tumour boards can save them golden hours of life?		
No	1 (1.3%)	-
May be	13 (16.3%)	3 (3.8%)
Yes	66 (82.5%)	77 (96.3%)
Are tumour boards cost-effective from the patient’s POV?		
No	10 (12.5%)	9 (11.3%)
May be	44 (55%)	7 (8.8%)
Yes	26 (32.5%)	64 (80%)
Are tumour boards a waste of time, energy, money and resources?		
No	79 (98.8%)	74 (92.5%)
Yes	1 (1.3%)	6 (7.5%)
Can tumour boards aid in the research and development?		
No	3 (3.8%)	-
Yes	77 (96.3%)	80 (100%)
Can tumour boards improve the mortality rate of cancer patients?		
No	19 (23.8%)	-
May be	-	4 (5%)
Yes	61 (76.3%)	76 (95%)
Are tumour boards mandatory?		
No	34 (42.5%)	25 (31.3%)
Yes	46 (57.5%)	55 (68.8%)
According to you, should tumour boards be made mandatory?		
No	3 (3.8%)	1 (1.3%)
Yes	77 (96.3%)	79 (98.8%)
Is it necessary to have a site-specific tumour board?		
No	18 (22.5%)	1 (1.3%)
Yes	62 (77.5%)	79 (98.8%)
Is it important to document a tumour board’s recommendation?		
No	8 (10%)	-
Yes	72 (90%)	80 (100%)
What should be the frequency of tumour board?		
Daily	2 (2.5%)	1 (1.3%)
Weekly	49 (61.3%)	76 (95%)
Fortnightly	4 (5%)	2 (2.5%)
Monthly	25 (31.3%)	1 (1.3%)
A tumour board meeting must include?		
Pathologist	1 (1.3%)	-
Radiologist	-	-
Oncologist	-	-
Surgeon	-	-
All	79 (98.8%)	80 (100%)
According to you, how much can a doctor benefit from the tumour boards?		
Least effective	-	-
Neutral	33 (41.3%)	8 (10%)
Most effective	47 (58.8%)	72 (90%)
According to you, how much can a patient benefit from the tumour boards?		
Least effective	-	-
Neutral	14 (17.5%)	1 (1.3%)
Most effective	66 (82.5%)	79 (98.8%)

**Table 3.** Association of pre and post mock test.

Mock items mean scores	Overall score %	Overall impact %
Pretest	**66.5%**	**28%**
Posttest	**94.62%**	

## References

[1] Abbasi AN (2019). Tumor board saves lives–more evidence is emerging for the mandatory development of site specific multi-disciplinary teams. Nat J Health Sci.

[2] O'Brien MP, Jungbluth N, Montecalvo J (2020). Advancing precision medicine education through a mock molecular tumor board in an academic medical center. J Cancer Educ.

[3] Mattes MD, Patel KR, Burt LM (2016). A nationwide medical student assessment of oncology education. J Cancer Educ.

[4] Oskvarek J, Braunstein S, Farnan J (2016). Medical student knowledge of oncology and related disciplines: a targeted needs assessment. J Cancer Educ.

[5] Mattes MD, Gerbo R, Dattola RM (2017). Tumor board shadowing for medical students as a means of early exposure to multidisciplinary oncology education. J Am Coll Radiol.

[6] Tsui JM, Grewal NKS, Sivapragasam M (2019). Tumor board shadowing: a unique approach for integrating radiation oncologists into general medical student education. Int J Radiat Oncol Biol Phys.

[7] Barnsteiner JH, Disch JM, Hall L (2007). Promoting interprofessional education. Nurs Outlook.

[8] Lee B, Kim K, Choi JY (2017). Efficacy of the multidisciplinary tumor board conference in gynecologic oncology: a prospective study. Medicine.

[9] Specchia ML, Frisicale EM, Carini E (2020). The impact of tumor board on cancer care: evidence from an umbrella review. BMC Health Serv Res.

[10] McKillip RP, Hahn OM, Bartkowiak B (2019). Implementation of a novel medical school multidisciplinary and interprofessional oncology curriculum: a mixed method study. J Cancer Educ.

[11] Nair BT (2019). Role play–an effective tool to teach communication skills in pediatrics to medical undergraduates. J Educ Health Promot.

[12] Mäurer I, Drescher R, Hammersen J (2023). Development and implementation of a student tumor board as a teaching format for medical students. J Cancer Res Clin Oncol.

[13] Qidwai W (2017). Growing disease burden in Pakistan: status, challenges, and opportunities. J Coll Physici Surg Pak.

[14] El Saghir NS, El-Asmar N, Hajj C (2011). Survey of utilization of multidisciplinary management tumor boards in Arab countries. Breast.

[15] Brandão M, Guisseve A, Bata G (2021). Survival impact and cost-effectiveness of a multidisciplinary tumor board for breast cancer in Mozambique, sub-Saharan Africa. Oncologist.

[16] Amgad M, Shash E, Gaafar R (2012). Cancer education for medical students in developing countries: where do we stand and how to improve?. Crit Rev Oncol/Hematol.

[17] Barrett WL, Aron BS, Breneman JC (2001). Clinical oncology clerkship for third‐year medical students. J Cancer Educ.

[18] Brower JV, Blitzer GC, Vapiwala N (2021). Declining medical student interest in radiation oncology: wake-up call with a silver lining?. Int J Radiat Oncol Biol Phys.

[19] Khan AM, Bhatti IA, Ali M (2021). Tumor board establishment; standpoint of our future doctors. Educ Res (IJMCER).

[20] Hopkins SE, Vidri RJ, Hill MV (2022). A virtual tumor board platform: a way to enhance decision-making for complex malignancies. J Surg Res.

